# Federated machine learning for predicting acute kidney injury in critically ill patients: a multicenter study in Taiwan

**DOI:** 10.1007/s13755-023-00248-5

**Published:** 2023-10-09

**Authors:** Chun-Te Huang, Tsai-Jung Wang, Li-Kuo Kuo, Ming-Ju Tsai, Cong-Tat Cia, Dung-Hung Chiang, Po-Jen Chang, Inn-Wen Chong, Yi-Shan Tsai, Yuan-Chia Chu, Chia-Jen Liu, Cheng-Hsu Chen, Kai-Chih Pai, Chieh-Liang Wu

**Affiliations:** 1 Institute of Emergency and Critical Care Medicine, National Yang-Ming Chiao Tung University, Taipei, Taiwan; 2 Nephrology and Critical Care Medicine, Department of Internal Medicine and Critical Care Medicine, Taichung Veterans General Hospital, Taichung, Taiwan; 3 Department of Critical Care Medicine, MacKay Memorial Hospital, Taipei, Taiwan; 4 Division of Pulmonary and Critical Care Medicine, Department of Internal Medicine, School of Medicine, College of Medicine, Kaohsiung Medical University, Kaohsiung, Taiwan; 5 Division of Critical Care Medicine, Department of Internal Medicine, National Cheng Kung University Hospital, College of Medicine, National Cheng Kung University, Tainan, Taiwan; 6 Department of Critical Care Medicine, Taipei Veterans General Hospital, Taipei, Taiwan; 7 Department of Information Technology, MacKay Memorial Hospital, Taipei, Taiwan; 8 Department of Diagnostic Radiology, National Cheng Kung University Hospital, College of Medicine, National Cheng Kung University, Tainan, Taiwan; 9 Department of Information Technology, Taipei Veterans General Hospital, Taipei, Taiwan; 10 Division of Nephrology, Department of Internal Medicine, Taichung Veterans General Hospital, Taichung, Taiwan; 11 College of Engineering, Tunghai University, Taichung, Taiwan; 12 College of Medicine, National Chung Hshin University, Taichung, Taiwan

**Keywords:** Acute kidney injury (AKI), Federated learning (FL), Prediction model

## Abstract

**Purpose:**

To address the contentious data sharing across hospitals, this study adopted a novel approach, federated learning (FL), to establish an aggregate model for acute kidney injury (AKI) prediction in critically ill patients in Taiwan.

**Methods:**

This study used data from the Critical Care Database of Taichung Veterans General Hospital (TCVGH) from 2015 to 2020 and electrical medical records of the intensive care units (ICUs) between 2018 and 2020 of four referral centers in different areas across Taiwan. AKI prediction models were trained and validated thereupon. An FL-based prediction model across hospitals was then established.

**Results:**

The study included 16,732 ICU admissions from the TCVGH and 38,424 ICU admissions from the other four hospitals. The complete model with 60 features and the parsimonious model with 21 features demonstrated comparable accuracies using extreme gradient boosting, neural network (NN), and random forest, with an area under the receiver-operating characteristic (AUROC) curve of approximately 0.90. The Shapley Additive Explanations plot demonstrated that the selected features were the key clinical components of AKI for critically ill patients. The AUROC curve of the established parsimonious model for external validation at the four hospitals ranged from 0.760 to 0.865. NN-based FL slightly improved the model performance at the four centers.

**Conclusion:**

A reliable prediction model for AKI in ICU patients was developed with a lead time of 24 h, and it performed better when the novel FL platform across hospitals was implemented.

**Supplementary Information:**

The online version contains supplementary material available at 10.1007/s13755-023-00248-5.

## Introduction

 Acute kidney injury (AKI) is a potentially life-threatening clinical syndrome with no effective treatment other than supportive care and dialysis [1]. The prevalence of AKI is approximately 30–60% for critically ill patients within 7 days of admission to the intensive care unit (ICU) [2]. Moreover, AKI is associated with higher rates of in-hospital mortality and long-term chronic kidney disease. Therefore, timely diagnosis and early awareness of AKI are crucial for its management [3].

A practical and concise AKI prediction model may reduce the burden of preventable and treatable AKI events. Recently, the use of deep learning and machine learning techniques for predicting AKI in critically ill patients has been increasing [4–6]. However, practical, generalizable, externally validated, and robust prediction models are relatively uncommon [7]. To develop an unbiased and generalized model, data from all target populations must be included.

A multicenter study is a potential solution for this issue. Currently, these multisite collaborations use centralized learning (CL), whereby data from different locations are shared in a centralized location following inter-site agreements [8]. The most extensive multisite collaboration to establish an AKI prediction model using CL was conducted by Tomasev et al. [9] who used a dataset of 703,782 patients from 172 inpatient and 1062 outpatient sites of the United States Department of Veterans Affairs. However, such data centralization cannot always be accomplished because sharing data outside each institute poses privacy and safety challenges [10].

Federated learning (FL), a framework to deal with possible data leakage issues in multicenter studies, has been put forth and gained widespread attention in various therapeutic fields recently. FL trains prediction models across multiple databases without the need to share or access individual data points. That said, unlike CL, it requires no centralized data warehouse infrastructure [8, 11, 12]. This method not only tackles the difficulties in gathering raw data from various hospital sources, but also ensures that patient privacy is protected [11, 12]. While implementing a FL framework in the healthcare fields, a cloud-based platform was built, allowing each to independently train their models using their local datasets. These hospital-specific models were then uploaded to the platform, and parameter adjustments were made based on the training outcomes [11, 12]. This approach facilitated the creation of generalizable models, e.g., a model capable of predicting AKI, across different hospital’s datasets while maintaining individual patient’s privacy.

Although researchers have focused on constructing appropriate machine-learning models for AKI prediction; however, the applications of most of their approaches are constrained by their data centralization nature. After an extensive literature search, it is found that few prior studies have successfully achieved an accurate model for AKI prediction based on decentralized medical data across different institutions. Accordingly, to address the aforementioned disadvantage of CL, there is an urgent need to establish a machine-learning model for AKI prediction in the ICU and construct a FL platform to determine if an aggregated FL model can outperform a single institute-trained model.

## Methods

### Ethics statement

This study was conducted in accordance with the ethical principles of the World Medical Association Declaration of Helsinki and the International Conference on Harmonization Good Clinical Practice Guidelines. The Institutional Research Board (IRB) of Taichung Veterans General Hospital (TCVGH) approved the study (TCVGH-IRB no. SE21473A) and waived the requirement for informed consent. The validation cohort at each hospital was approved by its own IRB (MMH-IRB No. 21MMHIS367e; KMUH-IRB No. E(I)-20,210,340; NCKUH-IRB No. A-ER-110-483; VGHTPE-IRB No. 2021-12-004BC).

### Dataset used to develop the AKI prediction model in critically ill patients

To build the machine-learning model for AKI prediction in critically ill patients, we extracted data from the critical care database of TCVGH, a medical center in the second largest metropolitan area in Taiwan, between 2015 and 2020. The dataset included all the consecutive patients admitted to the adult ICU and encompassed the comprehensive information during the ICU admissions, including 23 numerical and image data items, 339 features, and 33,508 ICU events (e.g., patients’ demographics, past medical history, and ICU severity scoring indices…, etc.).

Electronic medical records of the consecutive adult ICU patients in four other referral medical centers at different locations in Taiwan, i.e., Mackay Memorial Hospital (Taipei city, Taiwan), Taipei Veterans General Hospital (Taipei city, Taiwan), National Cheng Kung University Hospital (Tainan city, Taiwan), and Kaohsiung Medical University Hospital (Kaohsiung city, Taiwan) during 2018 to 2020 were utilized for the external validation and models training.

For patients who were repeatedly admitted to the ICUs, only the data of the first ICU admission are used. Exclusion criteria were patients younger than 20 years of age, with end-stage renal disease (ESRD) and receiving renal replacement therapy, had AKI occurred before the index ICU admission, underwent first hemodialysis within 24 h of ICU admission, and stayed in the ICU less than 30 h (Supplemental Fig. 1).

### AKI definition and labeling

AKI cases were labeled according to the Kidney Disease: Improving Global Outcomes (KDIGO) 2012 definition of AKI based on serum creatinine and urine output [13]. Briefly, KDIGO stage 1 AKI was characterized by increased serum creatinine to ≥ 0.3 mg/dL within 48 h, or an increase ≥ 1.5-times from the baseline value within 7 days, or urine output < 0.5 mL/kg/h for ≥ 6 h. Baseline serum creatinine was defined as the lowest value before the index ICU admission for patients transferred from the ward or the first available value for patients admitted directly from the emergency department, excluding serum creatinine values ≥ 4 mg/dL. Using the KDIGO criteria and their corresponding definitions for AKI severity, AKI was categorized as all-stage (KDIGO stages 1, 2, and 3), moderate and severe (KDIGO stages 2 and 3), and severe (KDIGO stage 3).

### Model development and evaluation

**Fig. 1:**
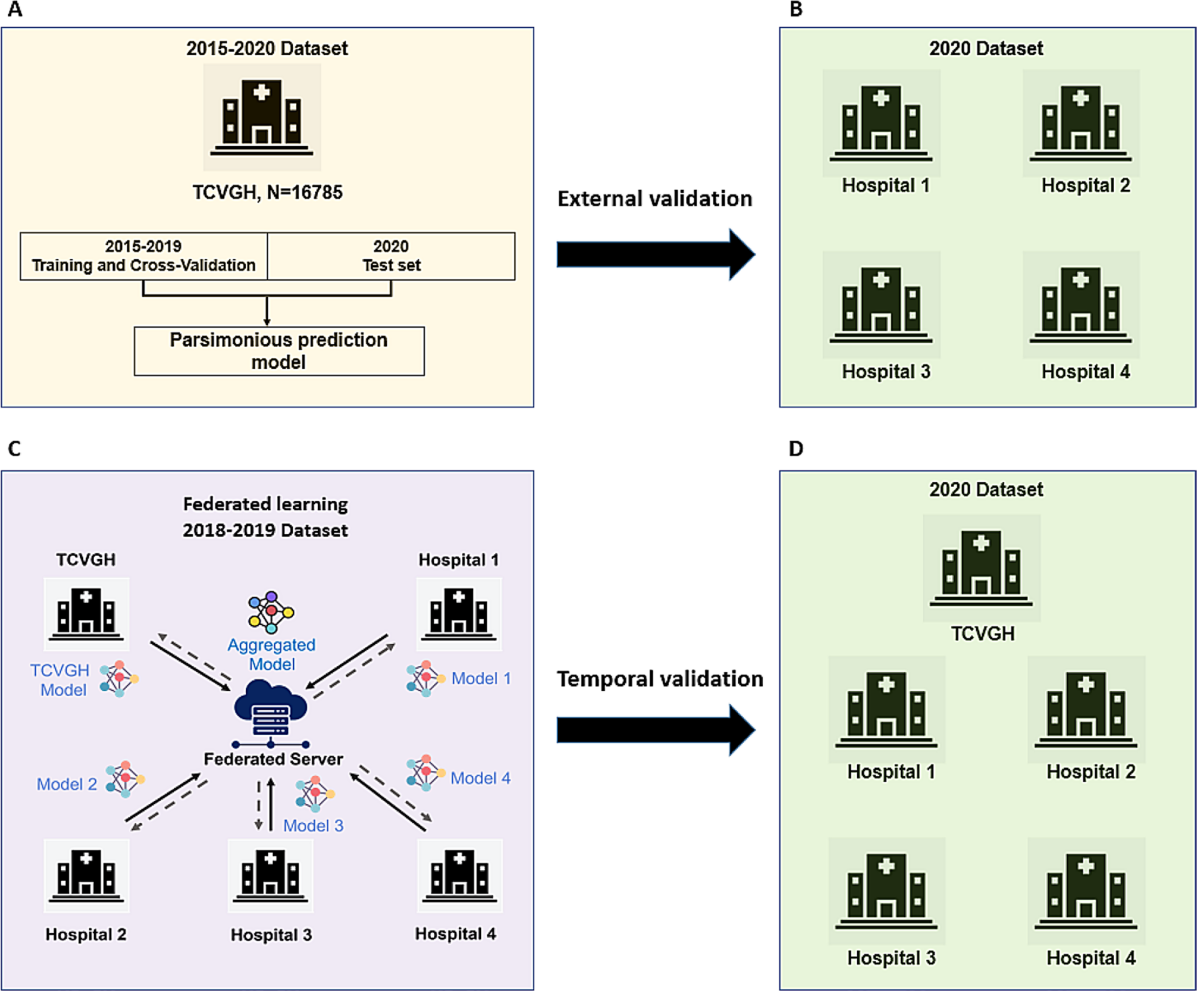
Illustration of the data workflow for model development, external validation, federated learning, and temporal validation. **A** Taichung Veterans General Hospital (TCVGH) model development. **B** External validation of the TCVGH model among four other hospitals. **C** Building the federated learning platform and creating the aggregated model. **D** Temporal validation of the aggregated model among the five hospitals

The TCVGH-AKI prediction model (Fig. 1A) was developed according to the transparent reporting of a multivariable prediction model for individual prognosis or diagnosis guidelines [14]. The study design used a 6-h feature window beginning from 24 h before the AKI event and a random 6-h feature window beginning from 30 h after ICU admission of non-AKI patients for machine learning (Supplemental Fig. 2). Sixty features were selected for model training, including age, vital signs, laboratory test results, and medications (Supplement Table 1). The details of the definition of the feature are provided in Supplement Table 2. Missing data were imputed with the mean for age, vital signs, and laboratory test results. Medications were input as categorical data, with 0 indicating no prescription and 1 indicating administration during the previous 7-day window. The missing proportion of the selected features in the TCVGH cohort is shown in Supplement Table 4. We applied four different machine learning models, eXtreme gradient boosting (XGBoost), neural network, random forest, and logistic regression, to the training cohort and performed five-fold cross-validation to build the initial prediction model. The rationale behind selecting these four algorithms is as follows: XGBoost: It efficiently handles missing data, incorporates regularization, and captures both linear and non-linear relationships [15]; Neural network: Particularly in deep learning, it automatically learns intricate patterns from diverse data sources, making them adept at predicting AKI onset using varied predictors [16]; Random forest: Functioning as an ensemble learner, it excels with large datasets, estimates missing values, and ranks variable importance [17]. In ICU data analysis it identifies critical AKI predictors that require vigilant monitoring. Logistic regression: Simple and interpretable, logistic regression calculates outcome probabilities. It is a valuable baseline model for binary AKI prediction, especially in clinical contexts. These four algorithms achieve a balance between simplicity and complexity, as well as between interpretability and performance. By comparing their performances, we aimed to identify the best model that balances accuracy and clinical interpretability for predicting AKI in ICU patients.

Model performance was evaluated using the area under the receiver-operating characteristic (AUROC) curve, calibration, and decision curve analysis [18, 19]. The Shapley Additive Explanations (SHAP) values were used to analyze the feature importance. The Platt scaling method was used for model calibration. We used the least absolute shrinkage and selection operator method to develop a parsimonious model with fewer features to improve its generalizability to other hospitals.

### External validation of the model

To ensure data privacy and consistency across different medical centers, TCVGH established a protocol for data cleaning and feature definition and provided a programming code package for alliance hospitals to process their data. These included automatic data cleaning, missing data imputation, and applying four parsimonious prediction model algorithms for external validation at the each of the other four hospitals. The characteristics of the AKI versus non-AKI groups and the missing proportion of selected features of the 2018–2020 cohort of five medical centers are provided in Supplement Tables 5 and 6, respectively.

### Development of an aggregated FL model

We developed an FL platform across the five hospitals: Taichung Veterans General Hospital (TCVGH), Mackay Memorial Hospital (MMH), Taipei Veterans General Hospital (TVGH), National Cheng Kung University Hospital (NCKU) and Kaohsiung Medical University Hospital (KMUH) (Fig. 1B). The 2018 to 2019 datasets of the five hospitals were used to develop an FL-based aggregated model. To fairly compare the performances of the aggregated FL and the parsimonious TCVGH models, they were tested using the individual hospital’s 2020 dataset. The same features used in the TCVGH parsimonious model were selected for training the FL model. At each participating hospital, the dataset was split into 80% for model training and 20% for internal validation using a neural network. The number of participating hospitals, K, was set to five, and network connectivity among the hospitals was confirmed before initiating FL. The number of federated rounds was set to 30, with two local training epochs per round at each hospital. The batch size was set to 32, and the number of local training iterations depended on the dataset size at each hospital. Using the Adam optimizer, the learning rate was set to 1 × 10^−2^ for both local learning and FL. Each hospital selected the best local model during the FL process by tracking its performance among the internal validation cohort. After each FL round, the central server determined the best-aggregated model based on the average validation scores from each hospital. When the FL was completed, the final best-aggregated model was evaluated among the temporal validation cohort at each hospital.

### Statistical analysis

The demographic and clinical characteristics of patients with and without AKI are presented as medians with interquartile ranges for continuous variables and as numbers (n) and proportions (%) for categorical variables. We used the Mann–Whitney *U* test to examine differences between groups for continuous data and the chi-square test for categorical data. P < 0.05 indicated statistical significance. All data processing and statistical analyses were performed using Python version 3.10.2.

## Results

### Derivation cohort description

The TCVGH cohort consisted of 13,861 ICU admissions from 2015 to 2019; 30.9% of the cohort had AKI and a 64.8% were male. Table 1 compares the demographic data of the AKI and non-AKI groups. Patients in the AKI group were older (70 years vs. 63 years) and had higher Acute Physiology and Chronic Health Evaluation II scores (26 vs. 20), higher Sequential Organ Failure Assessment scores (8 vs. 6), and a higher rate of vasopressor use (46.1% vs. 22.6%). In the AKI group, 30.2% received hemodialysis for the first time during ICU admission. The AKI group had poorer outcomes than the non-AKI group, including longer hospital stays (13.8 days vs. 4.3 days) and higher in-hospital mortality rates (41.0% vs. 6.2%). The 2020 TCVGH data were not used during model development to prevent overfitting, and their demographics had distributions similar to those of the derivation cohort.

**Table 1:** Characteristics of the AKI and non-AKI groups of the TCVGH derivation and internal validation cohorts

Demographics	Derivation cohort		*p-*value	Validation cohort		*p-*value
	AKI	Non-AKI		AKI	Non-AKI	
	N = 4287	N = 9574		N = 849	N = 2022	
Age (years)	70 (59–81)	63 (52–74)	< 0.001	69 (58–79)	63 (51–74)	< 0.001
Sex (male), n (%)	2700 (63)	6288 (65.7)	0.002	515 (60.7)	1274 (63)	0.236
BMI (kg/m^2^)	24.1 (21.4–27.2)	23.7 (21–26.5)	< 0.001	24.6 (21.7–27.9)	23.9 (21.2–26.7)	< 0001
Comorbidity and severity						
CCI	2 (1–5)	1 (0–3)	< 0.001	3 (1–5.3)	1 (0–3)	< 0.001
APACHE II score	26 (21–30)	20 (15–25)	< 0.001	24 (20–29)	19 (15–24)	< 0.001
SOFA score	8 (6–11)	6 (4–8)	< 0.001	8 (6–11)	6 (4–8)	< 0.001
Vasopressor, n (%)	1975 (46.1)	2161 (22.6)	< 0.001	403 (47.5)	435 (21.5)	< 0.001
Ventilator, n (%)	794 (18.5)	1382 (14.4)	< 0.001	249 (29.3)	407 (20.1)	< 0.001
Nephrotoxic medication						
NSAID COX-1 inhibitor	295 (6.9)	1065 (11.1)	< 0.001	73 (8.6)	266 (13.2)	< 0.001
NSAID COX-2 inhibitor	130 (3)	399 (4.2)	0.001	30 (3.5)	146 (7.2)	< 0.001
Vancomycin	465 (10.8)	919 (9.6)	0.024	105 (12.4)	208 (10.3)	0.103
Gentamicin	181 (4.2)	430 (4.5)	0.475	43 (5.1)	58 (2.9)	0.004
Colistin	171 (4)	39 (0.4)	< 0.001	23 (2.7)	5 (0.2)	< 0.001
Amphotericin B	57 (1.3)	24 (0.3)	< 0.001	9 (1.1)	8 (0.4)	0.034
Clinical data						
WBC count (/µL)	10,650 (7530–14,897.5)	9910 (7510–13,010)	< 0.001	10,490 (7740–14,650)	9940 (7515–13,067.5)	0.002
Hemoglobin (g/dL)	9.7 (8.7–11.3)	11.1 (9.6–12.9)	< 0.001	9.6 (8.4–11.2)	11.2 (9.7–12.9)	< 0.001
Platelet (10^3^/µL)	161 (92–243)	200 (146–263)	< 0.001	165 (92–245.8)	199 (144–261)	< 0.001
AST (mg/dL)	46 (28–80)	32 (22–56)	< 0.001	47 (29–79)	30 (21–55)	< 0.001
ALT (mg/dL)	29 (17–55)	25 (16–46)	< 0.001	32 (17–60)	25 (15–48)	< 0.001
Total bilirubin (mg/dL)	0.7 (0.5–1.5)	0.6 (0.4–1)	< 0.001	0.7 (0.4–1.5)	0.6 (0.4–0.9)	< 0.001
PT (seconds)	12.4 (11.1–14.8)	10.8 (10.1–11.9)	< 0.001	12.4 (11.3–14.7)	11.2 (10.5–12.3)	< 0.001
BUN (mg/dL)	31 (19–52)	17 (12–24)	< 0.001	30 (19–50)	18 (13–25)	< 0.001
Serum creatinine (mg/dL)	1.2 (0.8–2.1)	0.8 (0.6–1.1)	< 0.001	1.3 (0.8–2)	0.8 (0.7–1.1)	< 0.001
Serum Lactate (mg/dL)	19 (9.5–36.2)	13 (8.6–23.1)	< 0.001	14.5 (8.9–33.1)	10.2 (7.9–19.4)	< 0.001
24-h Urine output (mL)	1300 (730–2050)	2500 (1810–3420)	< 0.001	1320 (738.8–2102.0.5)	2542.5 (1830–3400)	< 0.001
Outcome						
Hemodialysis, n (%)	1295 (30.2)	0 (0)	< 0.001	249 (29.3)	0 (0)	< 0.001
ICU length of stay (days)	13.8 (6.9–23.6)	4.4 (2.7–8.8)	< 0.001	15.1 (7.4–25)	4.2 (2.6–8.9)	< 0.001
Hospital mortality, n (%)	1758 (41)	598 (6.2)	< 0.001	356 (41.9)	144 (7.1)	< 0.001

Data are presented as median (interquartile range) or number (%). *AKI* acute kidney injury, *ALT* alanine aminotransferase, *APACHE II* acute physiology and chronic health evaluation II, *AST* aspartate aminotransferase, *BMI* Body Mass Index, *BUN* blood urea nitrogen, *CCI* Charlson Comorbidity Index, *ICU* intensive care unit, *NSAID COX* non-steroid anti-inflammatory drug cyclooxygenase, *PT* prothrombin time, *SOFA* sequential organ failure assessment, *TCVGH* Taichung Veterans General Hospital, *WBC* white blood cell

Specifically, the severity of AKI is also documented in Supplement Table 10. Notably, around 30% of AKI patients are in the most severe stage requiring dialysis, indicating the critical nature of their condition and the potential need for intensive interventions such as dialysis. These scores, indices, and additional data emphasize the depth of the information captured in our study, providing a comprehensive understanding of the patients’ health status and the criticality of the disease.

### Performance of the full and parsimonious models

**Fig. 2:**
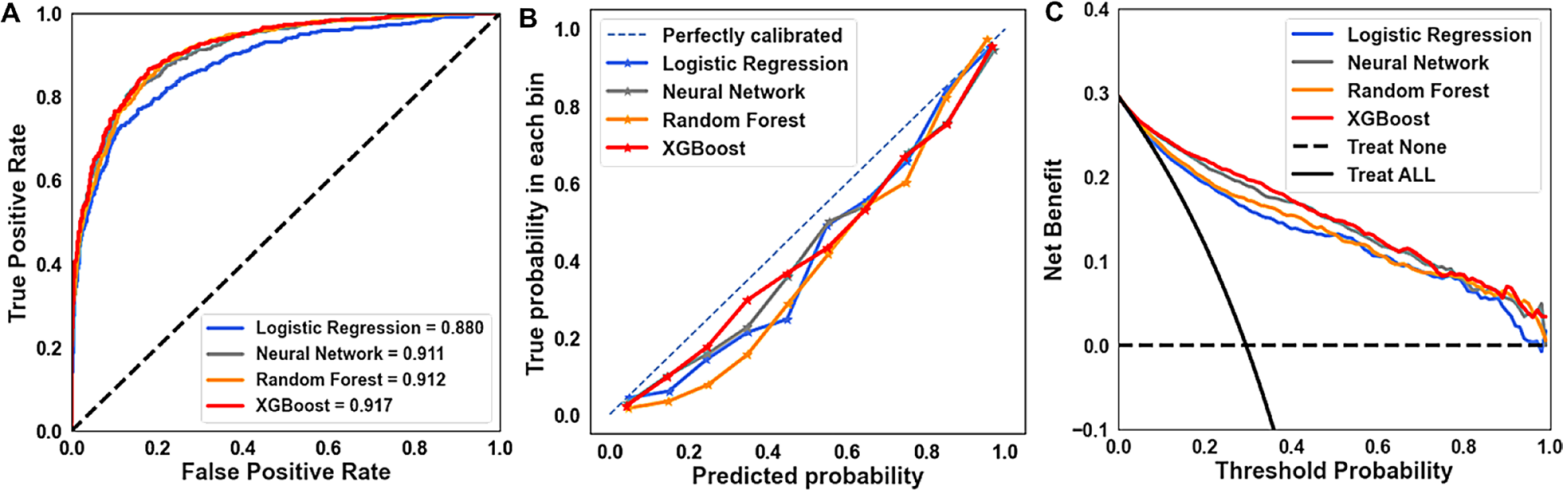
Taichung Veterans General Hospital (TCVGH) parsimonious model performance using the temporal validation dataset. **A** Receiver-operating characteristic (ROC) curve. **B** Calibration. **C** Decision curve analysis

**Fig. 3:**
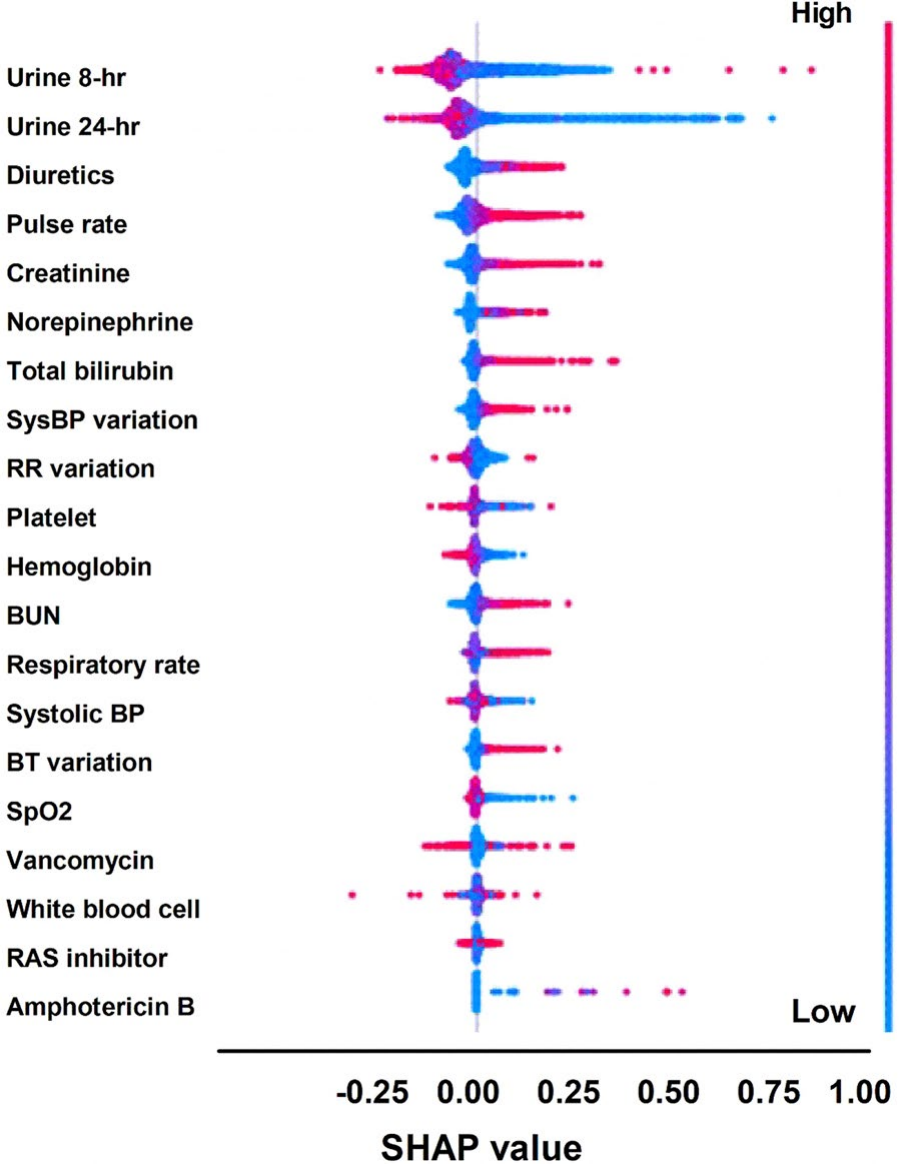
Shapley Additive Explanations (SHAP) plot of the neural network classifier in the parsimonious model. *BP* blood pressure, *BT* body temperature, *BUN* blood urea nitrogen, *RAS* renin aldosterone system, *RR* respiratory rate, *SysBP* systolic blood pressure

Sixty features were initially selected to construct the full model. The performances of the four classifiers within the full and parsimonious models are shown in Table 2. In the full models, XGBoost, neural network, and random forest performed better than logistic regression, with an AUROC curve value of 0.905 to 0.928, accuracy of 0.839 to 0.867, and precision of 0.692 to 0.769. After applying the least absolute shrinkage and selection operator for feature selection, the final 21 features (Supplemental Table 7) were included to develop the parsimonious model. The performance of the XGBoost, neural network, and random forest classifiers with the parsimonious model revealed a similar AUROC curve value (0.911–0.917) compared to the full model (Table 2; Fig. 2A). The calibration plot showed that all classifiers tended to overestimate the AKI risk (Fig. 2B). Based on the decision curve analysis comparing the four classifiers, XGBoost and the neural network had a higher net benefit across different probability thresholds, whereas logistic regression had the least net benefit (Fig. 2C). The SHAP plot of the neural network models revealed that 8- and 24-h urine output, diuretic use, pulse, and creatinine were the main five features contributing to the model prediction (Fig. 3).

**Table 2:** Comparison of TCVGH model performance with 60 versus 21 features using four classifiers among the temporal validation cohort

Classifier	Features	Sensitivity	Specificity	Precision	Accuracy	AUROC curve
XGBoost	60	0.787	0.901	0.769	0.867	0.928
	21	0.769	0.889	0.744	0.853	0.917
Neural network	60	0.766	0.879	0.726	0.845	0.905
	21	0.764	0.888	0.742	0.852	0.911
Random forest	60	0.822	0.846	0.692	0.839	0.913
	21	0.817	0.845	0.688	0.837	0.912
Logistic regression	60	0.728	0.875	0.710	0.831	0.878
	21	0.731	0.880	0.719	0.836	0.880

*AUROC* area under the receiver-operating characteristic, *TCVGH* Taichung Veterans General Hospital

### External validation of the parsimonious TCVGH model

We applied the parsimonious neural network model to four medical centers for external validation (Fig. 1B). Supplemental Table 5 summarizes the demographics and distribution of the input features of the external datasets from the four hospitals. The prevalence of AKI varied from 24.9 to 67.2% among 2874 to 12,483 cases at the four hospitals. The prediction performance decreased from 0.911 to 0.812 to 0.865 at three hospitals with an incidence of AKI comparable to that at TCVGH. For the hospital with fewer cases and a higher AKI incidence, the performance decreased to an AUC of 0.760 (Table 3, Supplemental Fig. 3).

**Table 3:** External validation of the parsimonious model among four hospitals in Taiwan

	N	Sensitivity	Specificity	Precision	Accuracy	AUROC curve
1. MMH	12,483	0.758	0.817	0.581	0.802	0.865
2. KMUH	12,299	0.668	0.768	0.561	0.737	0.812
3. NCKU	10,768	0.661	0.818	0.715	0.754	0.825
4. TVGH	2874	0.547	0.823	0.859	0.640	0.760

*AUROC* area under the receiver-operating characteristic, *MMH* Mackay Memorial Hospital, *TVGH* Taipei Veterans General Hospital, *NCKU* National Cheng Kung University Hospital, *KMUH* Kaohsiung Medical University Hospital

### Comparison of the aggregated FL and parsimonious TCVGH models

**Fig. 4:**
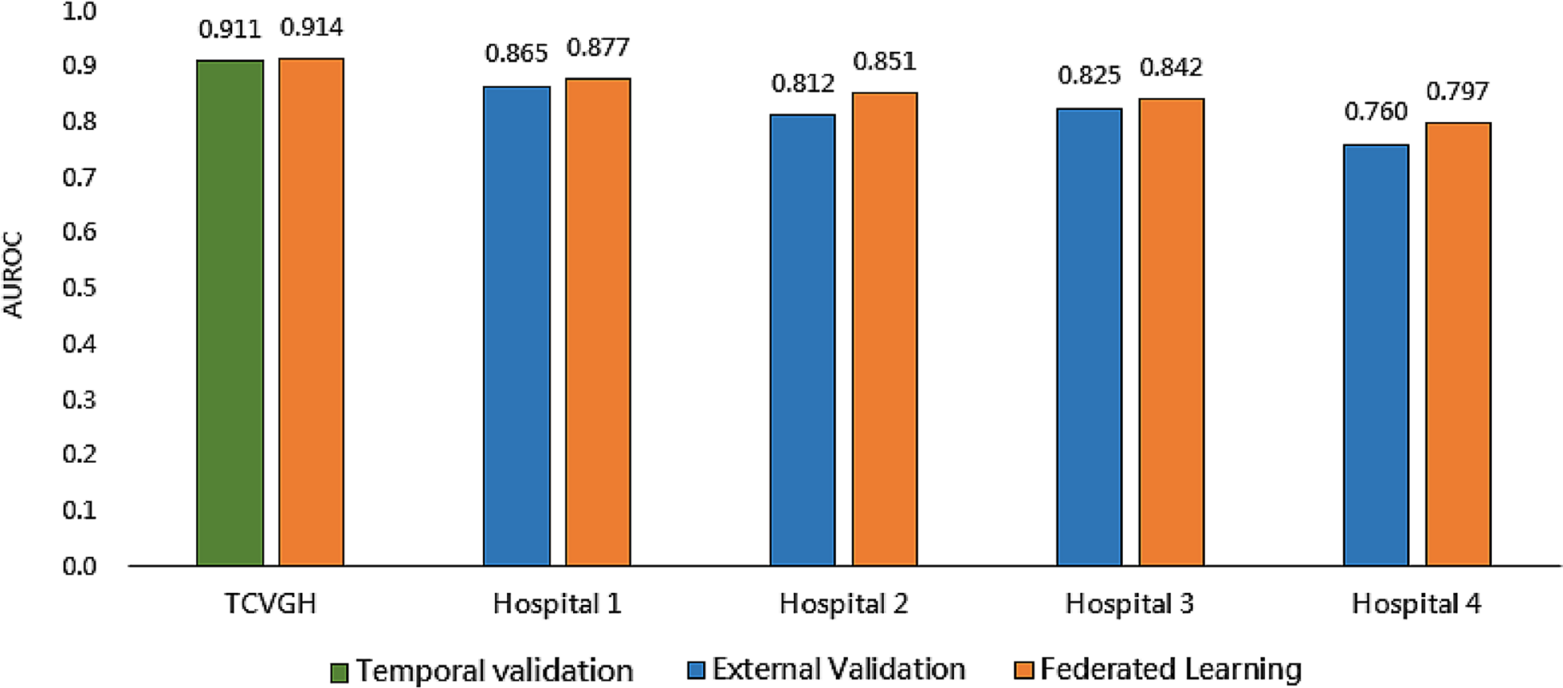
Performance comparison of the parsimonious Taichung Veterans General Hospital (TCVGH) model and the aggregated federated learning model. *AUROC* area under the receiver-operating characteristic

The aggregated FL model had statistically improved prediction performance compared to the parsimonious TCVGH model at the four hospitals, with the improvements in the AUROC curve ranging from 0.012 to 0.039. However, there was a slight improvement in the AUROC curve of 0.003 for TCVGH, but the difference was not significant (Fig. 4, Supplemental Table 8).

## Discussion

We developed a machine learning model with explainable features to predict KDIGO stages 1 to 3 AKI in adult ICU patients with a lead time of 24 h. Our model was externally validated at four independent medical centers in Taiwan and yielded promising results. We also established an FL platform enabling the creation of an aggregated model using model weight exchange among all five centers without sharing any raw data. This aggregated model outperformed the original TCVGH model for the remaining four hospitals.

AKI is a heterogeneous syndrome that can increase acute morbidity and mortality rates, thus affecting long-term cardiovascular and renal outcomes. Early diagnosis and treatment are essential to the prevention of long-term complications [20]. Electronic alerts have been suggested for the early diagnosis of AKI [21]. However, evidence of their benefits in ICUs [22] and general wards [23] is limited. More promising prediction models involving machine learning that were developed with different prediction windows and AKI severities have recently emerged. These models provide snapshot scores [24], moving windows [21], or continuous AKI prediction [25]. The prediction model generally has higher accuracy with a shorter prediction window (lead time) and more severe AKI.

Traditional machine learning-based AKI prediction models have achieved AUROC curve values from 0.75 to 0.90 according to internal validation studies and from 0.75 to 0.86 according to external validation studies [26, 27]. In this study, we assumed that predictors within a 6-h feature window before 24 h of an AKI event would be sufficient for machine learning to discriminate between AKI and non-AKI cases. A 24-h lead time for prediction also enables clinical usefulness by allowing clinicians to review high-risk patients for intervention.

Le et al. [6] developed a convolutional neural network for the AKI prediction model with a lead time of 24 h in the ICU based on the Medical Information Mart for Intensive Care III, which is a single-center dataset; their model showed AUC values of 0.834 and 0.867, predicting all-stage and stage 3 AKI, respectively. In comparison, our model demonstrated AUC values of 0.911 and 0.977 for predicting all-stage AKI and dialysis, respectively (Supplementary Table 9). Of note, AUC is chosen as it is widely used for binary classification tasks, like medical predictions. It gauges a model’s ability to differentiate cases regardless of the chosen threshold. A higher AUC means better prediction. In medicine, AUC is crucial to assess a model’s condition separation without tying it to a single threshold, aligning well with clinical considerations.

An explainable ML model is crucial to avoid black-box prediction and to create trust among clinicians. By applying the SHAP value, we found that decreased urine output, increased use of diuretics, higher heart rate, and increased serum creatinine levels were the main factors contributing to the prediction of AKI. Previous machine learning studies of AKI prediction in the ICU [26–28] have shown that serum creatine and urine output are usually among the main five features in the prediction models. Consistent with these studies, we found that the urine and serum creatinine levels used to define AKI were strong predictors of AKI in the ICU. Combining these two main features could streamline the complex model to a parsimonious one, making it easily applicable in hospitals. In contrast to other studies, we found that diuretics, which are used to increase urine output and are often prescribed to patients with fluid overload, are also a strong indicator of AKI. The feature importance analysis of our model mitigates the problem of black-box predictions and provides an explainable model for clinicians.

Only a few studies have externally validated the AKI prediction model in ICUs. Using the Medical Information Mart for Intensive Care IV dataset, Zhang et al. [26] developed an ensemble machine learning model to predict sepsis-associated AKI 12 to 48 h before its onset. The model was externally validated in the eICU Collaborative Research Database (eICU dataset), with an AUC of 0.774 to 0.788. The performance of our model among four external validation cohorts revealed an above-average performance, with an AUC of 0.760 to 0.865. However, the performance of our model deteriorated at the fourth hospital, which had a twofold higher prevalence of AKI than other hospitals (Supplemental Fig. 3). This finding suggests that changing outcome rates and shifting patient populations can affect the performance of the model.

Creating a generalizable model with healthcare data is challenging because of siloed data at individual hospitals and privacy concerns. Although external validation can test the generalizability of a model using more healthcare datasets, it cannot improve its performance without pooling raw data from healthcare institutes. Song et al. leveraged the United States PCORnet platform, demonstrated deterioration in the performance of the transported AKI prediction model among hospitalized patients across six independent health systems [7] and attributed it to the heterogeneity among the risk factors across populations. To address this issue, we created an FL framework to train an aggregated model with raw data stored at local institutions. Compared with the original TCVGH model, the aggregated FL model improved the prediction performance at the four external centers, possibly because of the ability of FL to capture more diversity and mitigate bias in homogeneous populations [8, 29]. Dang et al. [30] used the eICU dataset with 28 hospitals and 6641 patients to experiment with FL for AKI prediction; they designed a prediction model with 22 features, a 7-h feature window, and a 1-h prediction window and trained it with a neural network classifier. The local and aggregated average FL models showed AUROC curve values of 0.709 and 0.724, respectively. In contrast, our parsimonious model performed better (AUROC curve: 0.911), and the aggregated FL model showed an improved AUROC curve value of 0.012 to 0.039 at four external hospitals. Compared to a 1-h prediction window, the 24-h lead time of our model provides sufficient time for clinicians to intervene.

FL in healthcare is an emerging practical tool that enables effective collaboration among different hospitals to develop generalizable medical artificial intelligence (AI) [31, 32]. FL addresses the important barrier of data privacy in the global deployment of medical AI by allowing rapid model deployment while keeping private data securely stored at local hospitals [12, 33, 34]. One of the objective is to facilitate the implementation of the model across all ICUs by utilizing universally applicable parameters common to all ICUs and streamlining the parameter set for simplicity. By leveraging FL, the model derived has a higher likelihood of successful integration into clinical practice. Our study demonstrates the feasibility of the development of a generalizable medical AI using the FL platform without sharing raw data.

Our study had several limitations. First, our model was derived from and validated among Taiwanese datasets; therefore, it might not be generalizable to other ethnicities. Nonetheless, the original model could evolve into a more generalized one as more hospitals join the FL platform we built. Second, we used only 21 features to build the prediction model, and we used machine learning instead of more advanced tools, such as deep learning or ensemble machine learning. A complex model with more features or one trained using an advanced AI method might further improve the performance of the model. However, the trade-offs between simpler, faster, and more explainable models compared to complex and slower but more accurate models would depend on the interest in favoring practical application or academic research.

## Conclusions

Using datasets from five medical centers across Taiwan to develop and validate a parsimonious AKI prediction model with a lead time of 24 h in the ICU. An aggregated model built upon FL framework across the hospitals further improved the performance. This study shows that the adoption and integration of such a prediction model into clinical practice may be facilitated by applying FL based on the universally applicable features without sharing raw data of different institutions. Further research is still needed to translate this model to clinical outcomes of critically ill patients.

### Supplementary Information

Below is the link to the electronic supplementary material.

Supplementary material 1 (DOCX 360.5 kb)

## Data Availability

The data in this study were compiled from multiple sites across Taiwan using data-use agreements. Requests for data will require independent approvals from the TCVGH and partner institutions; requests can be made to the corresponding author. Given the promising performance of the algorithm, we are currently in the process of applying to the Taiwan Food and Drug Administration for approval of the TCVGH parsimonious model as a software/medical device and are unable to share the algorithm.
