# Supplementary material for: Federated machine learning for predicting acute kidney injury in critically ill patients: a multicenter study in Taiwan
Source: Health Inf Sci Syst. 2023 Oct 9;11(1):48. doi: 10.1007/s13755-023-00248-5 (PMC10562351; doi:10.1007/s13755-023-00248-5)
Supplement: Supplementary file 1 — Supplementary material 1 (DOCX 360.5 kb) [file 13755_2023_248_MOESM1_ESM.docx]

**Supplementary materials**

[Supplement Table 1 Catheter drainage is defined as urine output. 2](#_Toc143609099)

[Supplement Table 2 Initial 60 features input for TCVGH model development 3](#_Toc143609100)

[Supplement Table 3 Definition of clinical reasonable range for vital signs and LOINC/ATC code for laboratory data and medication 4](#_Toc143609101)

[Supplement Table 4 Proportion of missing features of the TCVGH cohort 5](#_Toc143609102)

[Supplement Table 5 Characteristics of the AKI versus non-AKI groups of the 2018-2020 cohort of five medical centers 6](#_Toc143609103)

[Supplement Table 5A: Hospital 6](#_Toc143609104)

[Supplement Table 5B: Hospital 2 8](#_Toc143609105)

[Supplement Table 5C: Hospital 3 10](#_Toc143609106)

[Supplement Table 5D: Hospital 4 12](#_Toc143609107)

[Supplement Table 5E: TCVGH 14](#_Toc143609108)

[Supplement Table 6 Proportion of missing features of the 2018-2020 cohort among five medical centers 16](#_Toc143609109)

[Supplement Table 7 Final 21 features input for TCVGH model and Federated Global model Training. 17](#_Toc143609110)

[Supplement Table 8 Performance comparison between TCVGH model and FL global model in five medical centers’ 2020 cohort 18](#_Toc143609111)

[Supplement Table 9 TCVGH model performance in predicting dialysis 24 hours in advance. 19](#_Toc143609112)

[Supplement Table 10 AKI stages of patients in each hospital. 20](#_Toc143609113)

[Supplement figure 1 Patient selection for model development in the TCVGH derivation cohort 21](#_Toc143609114)

[Supplement figure 2 Study design of the feature window and prediction window for machine learning AKI prediction 22](#_Toc143609115)

[Supplement figure 3 The association of AKI prevalence, case number, and model performance in a visualized graph 23](#_Toc143609116)

# Supplement Table 1 Catheter drainage is defined as urine output.

| Catheter item | System category |
| --- | --- |
| 3 Way Foley | Urinary bladder |
| Cystofix | Urinary bladder |
| Foley | Urinary bladder |
| Foley (Ileal-Conduit) | Urinary bladder |
| Nephrostomy Catheter | Renal system |
| PCN | Renal system |
| PCN SOFT Drainage Catheter (PCN) | Renal system |
| Silicone Foley | Urinary bladder |
| Suprapubic-Foley | Urinary bladder |
| Ureter Catheter | Renal system |

# Supplement Table 2 Initial 60 features input for TCVGH model development

| **Variable** | **Type** | **Number of features** | **Features** |
| --- | --- | --- | --- |
| **Age** | Continuous variable | 1 | Age |
| **Vital sign** | Mean/Variance | 14 | SBP, DBP, Pulse pressure, SpO2, Respiratory rate, pulse rate, Temperate, |
| **Urine** | Continuous variable | 3 | urine 8/16/24 hours |
| **Laboratory** | Latest observation | 7 | WBC count, Neutrophil, Hemoglobulin, Platelet count, BUN, Serum creatinine, Total Bilirubin |
| **Medication** | Prescribed within 7 days of ICU admission | 18 | Renin-angiotensin system inhibitors, diuretics, PPI, H2 receptor antagonists, NSAID-COX I inhibitors, NSAID COX II inhibitors, vasopressin, Norepinephrine, dopamine, epinephrine, dobutamine, vancomycin, Bactrim, Gentamicin, Amikacin, Colistin, Amphotericin B, Total medication |
| **Medication**  **time** | Time interval between last dose to prediction feature window | 17 | Renin-angiotensin system inhibitors, diuretics, PPI, H2 receptor antagonists, NSAID-COX I inhibitors COX II inhibitors, vasopressin, Norepinephrine, dopamine, epinephrine, dobutamine, vancomycin, Bactrim, Gentamicin, Amikacin, Colistin, Amphotericin B |

# Supplement Table 3 Definition of clinical reasonable range for vital signs and LOINC/ATC code for laboratory data and medication

| Category | Feature | Unit | Min | Max | LOINC/ATC code |
| --- | --- | --- | --- | --- | --- |
| Vital sign | SBP | mmHg | 0 | 300 |  |
|  | DBP | mmHg | 0 | 150 |  |
|  | Pulse pressure | mmHg | 0 | 200 |  |
|  | SpO2 | % | 0 | 100 |  |
|  | Respiratory rate | breath/min | 0 | 50 |  |
|  | Pulse rate | beat/min | 0 | 300 |  |
|  | Temperature | ℃ | 25 | 45 |  |
|  | | | Normal range | | LOINC |
| Lab | WBC count | /µL | 3500~11000 | | 26464-8  6690-2 |
|  | Hemoglobin | g/dL | 12~17.5 | | 718-7 |
|  | Platelet count | ×10^3^/µL | 150~400 | | 777-3 |
|  | BUN | mg/dL | 5~25 | | 3094-0 |
|  | Serum Creatinine | mg/dL | 0.3~1.4 | | 14682-9  2160-0 |
|  | Total Bilirubin | mg/dL | 0.2~1.2 | | 1975-2 |
|  |  | |  | | ATC code |
| Medication | Renin-angiotensin system inhibitors | |  | | First 3 number=C09 |
|  | Diuretics | |  |  | First 3 number=C03  Exclude CO3XA01 |
|  | Norepinephrine | |  |  | C01CA03 |
|  | Colistin | |  |  | J01XB01 |
|  | Amphotericin B | |  |  | J02AA01 |
|  | Vancomycin | |  |  | J01XA01 |

# Supplement Table 4 Proportion of missing features of the TCVGH cohort

|  | **2015-2019(Derivation)**  **N=13861** | **2020(Trending validation)**  **N=2871** |
| --- | --- | --- |
| **Feature** | Missing  n (%) | Missing  n (%) |
| Vital signs |  | |
| Temperature | 316 (2.3) | 95 (3.3) |
| Pulse rate | 4 (0.0) | 1 (0.0) |
| Respiratory rate | 4 (0.0) | 1 (0.0) |
| SBP | 37 (0.3) | 5 (0.2) |
| DBP | 37 (0.3) | 5 (0.2) |
| Pulse pressure | 37 (0.3 | 5 (0.2) |
| SpO2 | 158 (1.1) | 32 (1.1) |
| Lab data |  | |
| BUN | 1163 (8.4) | 886 (30.9) |
| Serum creatinine | 208 (1.5) | 293 (10.2) |
| WBC count | 190 (1.4) | 54 (1.9) |
| Neutrophil | 1812 (13.1) | 1056 (36.8) |
| Hemoglobin | 184 (1.3) | 54 (1.9) |
| Platelet | 210 (1.5) | 378 (13.2) |
| Total bilirubin | 1874 (13.5) | 52 (1.8) |

Data are presented as numbers (%). Abbreviations: SBP, systolic blood pressure; DBP, diastolic blood pressure; SpO2, saturation of peripheral oxygen; WBC, white blood cell; BUN, blood urea nitrogen

# Supplement Table 5 Characteristics of the AKI versus non-AKI groups of the 2018-2020 cohort of five medical centers

## Supplement Table 5A: Hospital

|  | **2018-2020 cohort** | | |
| --- | --- | --- | --- |
|  | **AKI** | **Non-AKI** | *p* Value |
|  | N=3114 | N=9369 |  |
| Age (Median/IQR) | 70(59-80) | 65(53-76) | <0.001 * |
| Male（n, %） | 1200(38.5) | 3441(36.7) | 0.070 |
| **Medication** | | | |
| Renin-angiotensin system inhibitors（n, %） | 280(9.0) | 1323(14.1) | <0.001 * |
| Diuretics（n, %） | 1160(37.3) | 2124(22.7) | <0.001 * |
| PPI（n, %） | 1078(34.6) | 3316(35.4) | 0.432 |
| H2 receptor antagonists  （n, %） | 408(13.1) | 1776(19.0) | <0.001 * |
| NSAID COX-1inhibitor  （n, %） | 235(7.5) | 1041(11.1) | <0.001 * |
| NSAID COX-2 inhibitor  （n, %） | 16(0.5) | 57(0.6) | 0.549 |
| Vasopressin（n, %） | 15(0.5) | 11(0.1) | <0.001 * |
| Norepinephrine（n, %） | 289(9.3) | 338(3.6) | <0.001 * |
| Dopamine（n, %） | 227(7.3) | 395(4.2) | <0.001 * |
| Epinephrine（n, %） | 384(12.3) | 381(4.1) | <0.001 * |
| Dobutamine（n, %） | 13(0.4) | 13(0.1) | 0.003 |
| Vancomycin（n, %） | 81(2.6) | 126(1.3) | <0.001 * |
| Bactrim（n, %） | 24(0.8) | 27(0.3) | <0.001 * |
| Gentamicin（n, %） | 103(3.3) | 370(3.9) | 0.104 |
| Amikacin（n, %） | 19(0.6) | 56(0.6) | 0.938 |
| Colistin（n, %） | 185(5.9) | 65(0.7) | <0.001 * |
| Amphotericin B（n, %） | 6(0.2) | 1(0.0) | 0.001 * |
| Total Med^†^(Median/IQR) | 2(0-3) | 1(0-2) | <0.001 * |
| **Vital signs** | | | |
| SBP(Median/IQR) | 118.3(105-134) | 128.0(114.2-141.5) | <0.001 * |
| MAP(Median/IQR) | 81.6(73.0-91.3) | 88.7(80.0-97.6) | <0.001 * |
| Pulse rate (Median/IQR) | 91.3(77.8-105.9) | 82.0(71.7-93.3) | <0.001 * |
| RR (Median/IQR) | 19.0(16.0-22.7) | 18.0(15.7-20.5) | <0.001 * |
| SpO2(Median/IQR) | 98.5(96.0-99.8) | 99.0(97.5-100.0) | <0.001 * |
| Temperature (Median/IQR) | 36.9(36.4-37.4) | 37.0(36.7-37.4) | <0.001 * |
| Urine 24(Median/IQR) | 920.0(134.3-1835.0) | 2240.0(1540.0-3070.0) | <0.001 * |
| **Lab data (Median/IQR1-3)** | | | |
| WBC count | 10900.0(7800.0-15200.0) | 10100.0(700.0-13400.0) | <0.001 * |
| Neutrophil | 81.0(72.0-87.8) | 79.4(70.0-86.0) | <0.001 * |
| Hemoglobin | 10.3(8.9-12.2) | 11.7(10.0-13.6) | <0.001 * |
| Platelet | 167.0(107.0-239.5) | 202.0(151.0-262.0) | <0.001 * |
| Albumin | 3.2(2.7-3.7) | 3.5(3.0-4.0) | <0.001 * |
| Total bilirubin | 1.0(0.7-1.6) | 0.9(0.6-1.3) | <0.001 * |
| Glucose | 148.5(114.0-206.0) | 139.0(114.0-183.0) | <0.001 * |
| BUN | 29.0(17.7-48.4) | 16.0(11.0-24.8) | <0.001 * |
| Serum creatinine | 1.4(0.9-2.2) | 0.9(0.7-1.2) | <0.001 * |
| PH_A | 7.43(7.37-7.48) | 7.44(7.40-7.48) | <0.001 * |

Data are presented as median (interquartile range) or number (%). Abbreviations: PPI, proton pump inhibitor; NSAID COX, non-steroid anti-inflammatory drugs cyclooxygenase; SBP, systolic blood pressure; MAP, mean arterial blood pressure; SpO2, saturation of peripheral oxygen; RR, respiratory rate; WBC, white blood cell; BUN, blood urea nitrogen; PH_A, pH value of arterial blood gas

^†^Total Med: Total medication means the total number of medications prescribed during the previous seven days of the feature window

* Statistically significant

## Supplement Table 5B: Hospital 2

|  | **2018-2020 cohort** | | |
| --- | --- | --- | --- |
|  | **AKI** | **Non-AKI** | *p* value |
|  | N=3866 | N=8433 |  |
| Age (Median/IQR) | 70(59-80) | 62(53-75) | <0.001 * |
| Male（n, %） | 2263(58.5) | 5391(63.9) | <0.001 * |
| **Medication** | | | |
| Renin-angiotensin system inhibitors（n, %） | 642(16.6) | 2035(24.1) | 0.001 |
| Diuretics（n, %） | 1673(43.3) | 1724(20.4) | <0.001 * |
| PPI（n, %） | 2070(52.5) | 3636(43.1) | <0.001 * |
| H2 receptor antagonists  （n, %） | 966(25) | 3612(42.8) | <0.001 * |
| NSAID COX-1inhibitor  （n, %） | 219(5.7) | 745(8.8) | 0.005 * |
| NSAID COX-2 inhibitor  （n, %） | 127(3.3) | 529(6.3) | <0.001 * |
| Vasopressin（n, %） | 167(4.3) | 77(0.9) | <0.001 * |
| Norepinephrine（n, %） | 1274(33) | 993(11.8) | <0.001 * |
| Dopamine（n, %） | 297(7.7) | 279(3.3) | <0.001 * |
| Epinephrine（n, %） | 577(14.9) | 393(4.7) | <0.001 * |
| Dobutamine（n, %） | 13(0.3) | 7(0.1) | 0.001 |
| Vancomycin（n, %） | 335(8.7) | 569(6.7) | <0.001 * |
| Bactrim（n, %） | 120(3.1) | 91(1.08) | <0.001 * |
| Gentamicin（n, %） | 84(2.2) | 439(5.2) | <0.001 * |
| Amikacin（n, %） | 26(0.7) | 21(0.2) | 0.013 * |
| Colistin（n, %） | 113(2.9) | 41(0.5) | 0.050 * |
| Amphotericin B（n, %） | 4(0.1) | 4(0.05) | 0.343 |
| Total Med^†^(Median/IQR) | 7(2-16) | 5 (2-10) | <0.001 * |
| **Vital signs** | | | |
| SBP(Median/IQR) | 125.5(111-145.3) | 132.5(117.8-148.5) | <0.001 * |
| MAP(Median/IQR) | 87.0(76.0-99.0) | 90.0(78.5-101.3) | <0.001 * |
| Pulse rate (Median/IQR) | 90.5(76.3-106.5) | 81.2(71.1-93.5) | <0.001 * |
| RR (Median/IQR) | 18.5(15.8-22) | 17.8(15.4-20.5) | <0.001 * |
| SpO2(Median/IQR) | 97.8(95.6-99.5) | 97.9(96-99.2) | <0.001 * |
| Temperature (Median/IQR) | 36.5(36-37.1) | 36.7(36.3-37.1) | <0.001 * |
| Urine 24(Median/IQR) | 1028.0(400.0-1648.0) | 2140.0(1440.0-1648.0) | <0.001 * |
| **Lab data (Median/IQR1-3)** | | | |
| WBC count | 10850.0(7660.0-15950.0) | 9990.0(7560.0-13280.0) | <0.001 * |
| Neutrophil | 84.7(75.6-91.1) | 81.8(72.9-88.4) | <0.001 * |
| Hemoglobin | 10.0(8.8-11.8) | 11.2(9.6-13) | <0.001 * |
| Platelet | 170.0(103.0-247.0) | 203.0(150.0-263.0) | <0.001 * |
| Albumin | 3.1(2.6-3.5) | 3.4(2.9-3.8) | <0.001 * |
| Total bilirubin | 1.1(0.7-2.0) | 0.9(0.6-1.4) | <0.001 * |
| Glucose | 146.0(118.0-193.0) | 132.0(110.0-167.0) | <0.001 * |
| BUN | 25.0(15.4-43.8) | 15.1(10.7-22.5) | <0.001 * |
| Serum creatinine | 1.1(0.7-1.9) | 0.8(0.6-1.1) | <0.001 * |
| PH_A | 7.41(7.35-7.47) | 7.42(7.38-7.46) | 0.01 |

Data are presented as median (interquartile range) or number (%). Abbreviations: PPI, proton pump inhibitor; NSAID COX, non-steroid anti-inflammatory drugs cyclooxygenase; SBP, systolic blood pressure; MAP, mean arterial blood pressure; SpO2, saturation of peripheral oxygen; RR, respiratory rate; WBC, white blood cell; BUN, blood urea nitrogen; PH_A, pH value of arterial blood gas

^†^Total Med: Total medication means the total number of medications prescribed during the previous seven days of the feature window

* Statistically significant

## Supplement Table 5C: Hospital 3

|  | **2018-2020 cohort** | | |
| --- | --- | --- | --- |
|  | **AKI** | **Non-AKI** | *p* value |
|  | N= 4165 | N= 6603 |  |
| Age (Median/IQR) | 69 (58-80) | 64 (53-75) | <0.001 * |
| Male（n, %） | 2536 (60.9) | 4240 (64.1) | 0.107 |
| **Medication** | | | |
| Renin-angiotensin system inhibitors（n, %） | 383 (9.2) | 909 (13.8) | <0.001 * |
| Diuretics（n, %） | 1602 (38.5) | 1621 (24.5) | <0.001 * |
| PPI（n, %） | 2295 (55.1) | 3582 (54.2) | 0.639 |
| H2 receptor antagonists  （n, %） | 263 (6.3) | 500 (7.6) | 0.021 |
| NSAID COX-1inhibitor  （n, %） | 76 (1.8) | 322 (4.9) | <0.001 * |
| NSAID COX-2 inhibitor  （n, %） | 22 (0.5) | 81 (1.2) | <0.001 * |
| Vasopressin（n, %） | 232 (5.6) | 77 (1.2) | <0.001 * |
| Norepinephrine（n, %） | 1649 (39.6) | 985 (14.9) | <0.001 * |
| Dopamine（n, %） | 457 (11.0) | 430 (6.5) | <0.001 * |
| Epinephrine（n, %） | 522 (12.5) | 232 (3.5) | <0.001 * |
| Dobutamine（n, %） | 628 (15.1) | 438 (6.6) | <0.001 * |
| Vancomycin（n, %） | 319 (7.7) | 383 (5.8) | <0.001 * |
| Bactrim（n, %） | 129 (3.1) | 86 (1.3) | <0.001 * |
| Gentamicin（n, %） | 40 (1.0) | 81 (1.2) | 0.207 |
| Amikacin（n, %） | 16 (0.4) | 144 (2.2) | <0.001 * |
| Colistin（n, %） | 50 (1.2) | 15 (0.2) | <0.001 * |
| Amphotericin B（n, %） | 19 (0.5) | 11 (0.2) | 0.006 * |
| Total Med^†^(Median/IQR) | 12 (2-39) | 4 (0-13) | <0.001 * |
| **Vital signs** | | | |
| SBP(Median/IQR) | 121.6 (108.2-137.4) | 130.9 (118.3-142.9) | <0.001 * |
| MAP(Median/IQR) | 82.0 (74.2-90.9) | 87.8 (80.2-95.2) | <0.001 * |
| Pulse rate (Median/IQR) | 93.2 (79.3-107.6) | 83.9 (73.4-95.3) | <0.001 * |
| RR (Median/IQR) | 17.7 (15.0-21.2) | 17.8 (15.3-20.3) | 0.186 |
| SpO2(Median/IQR) | 98.7 (96.9-99.7) | 98.8 (97.3-99.8) | <0.001 * |
| Temperature (Median/IQR) | 36.6 (36.1-37.2) | 36.7 (36.2-37.1) | 0.035 * |
| Urine 24(Median/IQR) | 1050.0(455.0-1800.0) | 1890.0(1370.0-2610.0) | <0.001 * |
| **Lab data (Median/IQR1-3)** | | | |
| WBC count | 10600.0 (7500.0-14900.0) | 10000.0(7600.0-13200.0) | <0.001 * |
| Neutrophil | 83.4 (74.8-89.1) | 81.4 (73.0-86.6) | <0.001 * |
| Hemoglobin | 10.7 (9.4-12.3) | 11.0 (9.9-12.6) | <0.001 * |
| Platelet | 171.0 (106.0-248.0) | 194.0 (137.0-260.0) | <0.001 * |
| Albumin | 3.1 (2.6-3.5) | 3.2 (2.8-3.7) | <0.001 * |
| Total bilirubin | 0.9 (0.5-1.8) | 0.8 (0.5-1.2) | <0.001 * |
| Glucose | 162.0 (127.0-220.8) | 151.0 (124.0-195.0) | <0.001 * |
| BUN | 27.0 (18.0-46.0) | 18.0 (12.0-27.0) | <0.001 * |
| Serum creatinine | 1.0(0.7-1.7) | 0.7 (0.5-1.0) | <0.001 * |
| PH_A | 7.44 (7.38-7.48) | 7.45(7.43-7.48) | <0.001 * |

Data are presented as median (interquartile range) or number (%). Abbreviations: PPI, proton pump inhibitor; NSAID COX, non-steroid anti-inflammatory drugs cyclooxygenase; SBP, systolic blood pressure; MAP, mean arterial blood pressure; SpO2, saturation of peripheral oxygen; RR, respiratory rate; WBC, white blood cell; BUN, blood urea nitrogen; PH_A, pH value of arterial blood gas

^†^Total Med: Total medication means the total number of medications prescribed during the previous seven days of the feature window

* Statistically significant

## Supplement Table 5D: Hospital 4

|  | **2018-2020 cohort** | | |
| --- | --- | --- | --- |
|  | **AKI** | **Non-AKI** | *p* Value |
|  | N= 1932 | N= 942 |  |
| Age (Median/IQR) | 71(60-83) | 70(58-83) | 0.084 |
| Male（n, %） | 722(37.4) | 327(34.7) | 0.165 |
| **Medication** | | | |
| Renin-angiotensin system inhibitors（n, %） | 59(3.1) | 20(2.1) | 0.152 |
| Diuretics（n, %） | 1149(59.5) | 455(48.3) | < 0.001 * |
| PPI（n, %） | 610(31.6) | 200(21.2) | < 0.001 * |
| H2 receptor antagonists  （n, %） | 66(3.4) | 33(3.5) | 0.904 |
| NSAID COX-1inhibitor  （n, %） | 85(4.4) | 29(3.1) | 0.089 |
| NSAID COX-2 inhibitor  （n, %） | 36(1.9) | 10(1.1) | 0.108 |
| Vasopressin（n, %） | 70(3.6) | 13(1.4) | 0.001* |
| Norepinephrine（n, %） | 694(35.9) | 185(19.6) | < 0.001 * |
| Dopamine（n, %） | 144(7.5) | 28(3) | < 0.001 * |
| Epinephrine（n, %） | 306(15.8) | 105(11.1) | 0.001 * |
| Dobutamine（n, %） | 12(0.6) | 3(0.3) | 0.411 |
| Vancomycin（n, %） | 45(2.3) | 8(0.8) | 0.006 * |
| Bactrim（n, %） | 54(2.8) | 20(2.1) | 0.286 |
| Gentamicin（n, %） | 9(0.5) | 2(0.2) | 0.521 |
| Amikacin（n, %） | 10(0.5) | 7(0.7) | 0.459 |
| Colistin（n, %） | 124(6.4) | 40(4.2) | 0.018 * |
| Amphotericin B（n, %） | 15(0.8) | 5(0.5) | 0.457 |
| Total Med^†^(Median/IQR) | 8(4-20) | 4(0-12) | < 0.001 * |
| **Vital signs** | | | |
| SBP(Median/IQR) | 119.1(103.4-139.9) | 130.4(112.4-150.4) | < 0.001 * |
| MAP(Median/IQR) | 78.8(69.8-90.9) | 82.2(67.3-94.7) | < 0.001 * |
| Pulse rate (Median/IQR) | 93.4(79.2-106.8) | 90.1(78.2-101.8) | < 0.001 * |
| RR (Median/IQR) | 17.1(14.6-20.5) | 18.0(15.0-21.1) | <0.001 * |
| SpO2(Median/IQR) | 97.5(95.6-99) | 97.7(96.2-99) | 0.497 |
| Temperature (Median/IQR) | 37.0(36.5-37.5) | 37.0(36.5-37.4) | 0.781 |
| Urine 24(Median/IQR) | 1190.0(530.0-2115.0) | 2150.0(1530.0-3040.0) | < 0.001 * |
| **Lab data (Median/IQR1-3)** | | | |
| WBC count | 9500.0(6200.0-14050.0) | 9500.0(6800.0-12600.0) | 0.001* |
| Neutrophil | 85.0(76.3-91.1) | 82.7(75-88.7) | 0.491 |
| Hemoglobin | 9.2(8.3-10.4) | 9.5(8.6-10.6) | 0.001 * |
| Platelet | 122.0(69-195) | 172.0(98.5-268.5) | < 0.001 * |
| Albumin | 2.9(2.6-3.3) | 3.0(2.6-3.4) | 0.065 |
| Total bilirubin | 1.1(0.6-2.7) | 0.6(0.4-1.3) | < 0.001 * |
| Glucose | 149.0(113.5-212.5) | 147.0(111-208) | 0.296 |
| BUN | 31.0(19.0-51.0) | 28.0(18.0-45.0) | < 0.001 * |
| Serum creatinine | 1.2(0.8-2.0) | 0.9(0.7-1.3) | < 0.001 * |
| PH_A | 7.44(7.39-7.48) | 7.47(7.44-7.50) | < 0.001 * |

Data are presented as median (interquartile range) or number (%). Abbreviations: PPI, proton pump inhibitor; NSAID COX, non-steroid anti-inflammatory drugs cyclooxygenase; SBP, systolic blood pressure; MAP, mean arterial blood pressure; SpO2, saturation of peripheral oxygen; RR, respiratory rate; WBC, white blood cell; BUN, blood urea nitrogen; PH_A, pH value of arterial blood gas

^†^Total Med: Total medication means the total number of medications prescribed during the previous seven days of the feature window

* Statistically significant

## Supplement Table 5E: TCVGH

|  | **2018-2020 cohort** | | |
| --- | --- | --- | --- |
|  | **AKI** | **Non-AKI** | *p* value |
|  | N=2654 | N=6292 |  |
| Age (Median/IQR) | 69[58-80] | 63[51-74] | <0.001 * |
| Male（n, %） | 1641(61.831) | 4069(64.669) | 0.011 |
| **Medication** | | | |
| Renin-angiotensin system inhibitors（n, %） | 589(22.2) | 2012(32.0) | <0.001 * |
| Diuretics（n, %） | 1807(68.1) | 2462(39.1) | <0.001 * |
| PPI（n, %） | 1939(73.1) | 4408(70.1) | 0.004 |
| H2 receptor antagonists  （n, %） | 278(10.5) | 896(14.2) | <0.001 * |
| NSAID COX-1inhibitor  （n, %） | 182(6.9) | 715(11.4) | <0.001 * |
| NSAID COX-2 inhibitor  （n, %） | 72(2.7) | 344(5.5) | <0.001 * |
| Vasopressin（n, %） | 173(6.5) | 51(0.8) | <0.001 * |
| Norepinephrine（n, %） | 1268(47.8) | 1283(20.4) | <0.001 * |
| Dopamine（n, %） | 322(12.1) | 523(8.3) | <0.001 * |
| Epinephrine（n, %） | 471(17.7) | 467(7.4) | <0.001 * |
| Dobutamine（n, %） | 79(3.0) | 127(2.0) | 0.006 |
| Vancomycin（n, %） | 277(10.4) | 595(9.5) | 0.153 |
| Bactrim（n, %） | 118(4.4) | 85(1.4) | <0.001 * |
| Gentamicin（n, %） | 117(4.4) | 178(2.8) | <0.001 * |
| Amikacin（n, %） | 22(0.8) | 23(0.4) | 0.005 |
| Colistin（n, %） | 88(3.3) | 18(0.3) | <0.001 * |
| Amphotericin B（n, %） | 33(1.2) | 20(0.3) | <0.001 * |
| Total Med^†^(Median/IQR) | 15(7-26) | 8(4-15) | <0.001 * |
| **Vital signs** | | | |
| SBP(Median/IQR) | 116.0(103.7-130.3) | 123.7(112.0-135.3) | <0.001 * |
| MAP(Median/IQR) | 83.3(75.24-92.67) | 88.9(80.9-97.0) | <0.001 * |
| Pulse rate (Median/IQR) | 91.7(78.3-106.33) | 80.3(70.7-91.7) | <0.001 * |
| RR (Median/IQR) | 19.0(16.3-22.7) | 17.8(15.8-20.0) | <0.001 * |
| SpO2(Median/IQR) | 98.0(95.8-99.7) | 98.3(97.0-99.7) | <0.001 * |
| Temperature (Median/IQR) | 36.5(36.0-37.0) | 36.5(36.1-36.9) | 0.975 |
| Urine 24(Median/IQR) | 1300.0(728.0-2050.0) | 2510.0(1810.0-3400.0) | <0.001 * |
| **Lab data (Median/IQR1-3)** | | | |
| WBC count | 10360.0(7510.0-14620.0) | 9830.0(7447.5-12930.0) | <0.001 * |
| Neutrophil | 84.2(76.7-89.7) | 81.8(73.3-88.1) | <0.001 * |
| Hemoglobin | 9.6(8.6-11.2) | 11.2(9.7-12.9) | <0.001 * |
| Platelet | 162.0(91.0-240.8) | 203.0(149.0-265.0) | <0.001 * |
| Albumin | 2.8(2.4-3.3) | 3.2(2.8-3.8) | <0.001 * |
| Total bilirubin | 0.7(0.4-1.6) | 0.6(0.4-0.9) | <0.001 * |
| Glucose | 153.0(115.0-210.0) | 129.0(103.0-172.0) | <0.001 * |
| BUN | 31.0(19.0-51.0) | 17.0(12.0-24.0) | <0.001 * |
| Serum creatinine | 1.25(0.8-1.98) | 0.81(0.64-1.05) | <0.001 * |
| PH_A | 7.42(7.37-7.47) | 7.43(7.39-7.47) | <0.001 * |

Data are presented as median (interquartile range) or number (%). Abbreviations: PPI, proton pump inhibitor; NSAID COX, non-steroid anti-inflammatory drugs cyclooxygenase; SBP, systolic blood pressure; MAP, mean arterial blood pressure; SpO2, saturation of peripheral oxygen; RR, respiratory rate; WBC, white blood cell; BUN, blood urea nitrogen; PH_A, pH value of arterial blood gas

^†^Total Med: Total medication means the total number of medications prescribed during the previous seven days of the feature window

* Statistically significant

# Supplement Table 6 Proportion of missing features of the 2018-2020 cohort among five medical centers

|  | **Hospital 1**  N=12483 | **Hospital 2**  N=12299 | **Hospital 3**  N=10768 | **Hospital 4**  N=2874 | **TCVGH**  N=8946 |
| --- | --- | --- | --- | --- | --- |
| **Feature** | Missing  n (%) | Missing  n (%) | Missing  n (%) | Missing  n (%) | Missing  n (%) |
| Vital signs |  | | | | |
| Temperature | 477 (3.8) | 954 (7.8) | 196 (1.8) | 77 (2.7) | 251 (2.8) |
| Pulse rate | 421 (3.4) | 949 (7.7) | 18 (0.2) | 15 (0.5) | 2 (0.0) |
| Respiratory rate | 426 (3.4) | 951 (7.7) | 18 (0.2) | 1 (0.0) | 2 (0.0) |
| SBP | 421 (3.4) | 1028 (8.4) | 22 (0.2) | 254 (8.8) | 18 (0.2) |
| DBP | 421 (3.4) | 1028 (8.4) | 22 (0.2) | 254 (8.8) | 18 (0.2) |
| Pulse pressure | 421 (3.4) | 1028 (8.4) | 22 (0.2) | 254 (8.8) | 18 (0.2) |
| SpO2 | 0 (0.0) | 0 (0.0) | 0 (0.0) | 37 (1.3) | 122 (1.4) |
| Lab data |  | | | | |
| Albumin | 5306 (42.5) | 3529 (28.7) | 4314 (40.1) | 476 (16.6) | 3107 (34.7) |
| BUN | 848 (6.8) | 431 (3.5) | 1150 (10.7) | 126 (4.4) | 801 (9.0) |
| Serum creatinine | 747 (6.0) | 412 (3.4) | 49 (0.5) | 106 (3.7) | 140 (1.6) |
| Glucose | 3111 (24.9) | 558 (4.5) | 1282 (11.9) | 268 (9.3) | 3451 (38.6) |
| WBC count | 886 (7.1) | 65 (0.5) | 76 (0.7) | 76 (2.7) | 135 (1.5) |
| Neutrophil | 944 (7.6) | 1965 (16.0) | 50 (0.5) | 256 (8.9) | 1144 (12.8) |
| Hemoglobin | 773 (6.2) | 65 (0.5) | 35 (0.3) | 255 (8.9) | 131 (1.5) |
| Platelet | 1162 (9.3) | 65 (0.5) | 54 (0.5) | 69 (2.4) | 142 (1.6) |
| PH_A | 4510 (36.1) | 1635 (13.3) | 1172 (10.9) | 256 (8.9) | 2096 (23.4) |
| Total bilirubin | 5524 (44.3) | 5082 (41.3) | 5849 (54.3) | 252 (8.8) | 939 (10.5) |

Data are presented as number (%). Abbreviations: SBP, systolic blood pressure; DBP, diastolic blood pressure; SpO2, saturation of peripheral oxygen; RR, respiratory rate; WBC, white blood cell; BUN, blood urea nitrogen; PH_A, pH value of arterial blood gas

# Supplement Table 7 Final 21 features input for TCVGH model and Federated Global model Training.

| **Variable** | **Type** | **Number of features** | **Features** |
| --- | --- | --- | --- |
| **Vital sign** | Mean/Variance | 9 | SBP, SBP variance, SpO2, Respiratory rate, Respiratory rate variance, Pulse rate, Temperature variance, urine 8 hours, urine 24 hours |
| **Laboratory** | Latest observation | 6 | WBC, Hemoglobulin, Platelet count, BUN, Serum creatinine, Total Bilirubin, |
| **Medication** | Prescribed within 7 days of ICU admission | 5 | Renin-angiotensin system inhibitors, diuretics, Norepinephrine, Colistin, Amphotericin B |
| **Medication**  **time** | Time interval between last dose to prediction feature window | 1 | Vancomycin |

# Supplement Table 8 Performance comparison between TCVGH model and FL global model in five medical centers’ 2020 cohort

|  | TCVGH model (2015-2019)  Classifier: Neural Network | | | | | FL aggregated model (2018-2019)  Classifier: Neural Network | | | | | Delong test | |
| --- | --- | --- | --- | --- | --- | --- | --- | --- | --- | --- | --- | --- |
|  | Sensitivity | Specificity | Precision | Accuracy | AUROC | Sensitivity | Specificity | Precision | Accuracy | AUROC | Delta  AUROC | *p* value |
| TCVGH | 0.764 | 0.888 | 0.742 | 0.852 | 0.911 | 0.762 | 0.897 | 0.756 | 0.857 | 0.914 | 0.003 | 0.199 |
| Hospital 1 | 0.758 | 0.817 | 0.581 | 0.802 | 0.865 | 0.749 | 0.847 | 0.622 | 0.823 | 0.877 | 0.012 | <0.001 |
| Hospital 2 | 0.668 | 0.768 | 0.561 | 0.737 | 0.812 | 0.664 | 0.854 | 0.669 | 0.796 | 0.851 | 0.039 | <0.001 |
| Hospital 3 | 0.661 | 0.818 | 0.715 | 0.754 | 0.825 | 0.652 | 0.859 | 0.761 | 0.774 | 0.842 | 0.018 | <0.001 |
| Hospital 4 | 0.547 | 0.823 | 0.859 | 0.640 | 0.760 | 0.547 | 0.867 | 0.891 | 0.655 | 0.797 | 0.037 | <0.001 |

# Supplement Table 9 TCVGH model performance in predicting dialysis 24 hours in advance.

|  | TCVGH model for dialysis prediction | | | | | | | | | |
| --- | --- | --- | --- | --- | --- | --- | --- | --- | --- | --- |
|  | 2015-2019 Derivation cohort | | | | | 2020 Temporal validation cohort | | | | |
| Classifier | Sensitivity | Specificity | Precision | Accuracy | AUROC | Sensitivity | Specificity | Precision | Accuracy | AUROC |
| XGBoost | 0.887  ±0.019 | 0.989  ±0.002 | 0.830  ±0.026 | 0.983  ±0.002 | 0.991  ±0.002 | 0.846 | 0.989 | 0.818 | 0.981 | 0.976 |
| Neural Network | 0.913  ±0.024 | 0.970  ±0.003 | 0.649  ±0.037 | 0.967  ±0.003 | 0.978  ±0.008 | 0.863 | 0.976 | 0.669 | 0.969 | 0.977 |
| Random Forest | 0.935  ±0.025 | 0.956  ±0.007 | 0.565  ±0.050 | 0.954  ±0.008 | 0.988  ±0.002 | 0.915 | 0.953 | 0.527 | 0.951 | 0.976 |
| Logistic Regression | 0.916  ±0.019 | 0.950  ±0.007 | 0.532  ±0.047 | 0.948  ±0.007 | 0.977  ±0.009 | 0.915 | 0.961 | 0.575 | 0.959 | 0.975 |

# Supplement Table 10 AKI stages of patients in each hospital.

|  | **Hospital** | | | | |
| --- | --- | --- | --- | --- | --- |
|  | MMH | NCKU | TCVGH | TVGH | KMUH |
| **Group** | n= 12,483 | n= 10,768 | n= 16,732 | n= 2,872 | n= 12,299 |
| **Non-AKI** | 9369 (75.1) | 6603 (61.3) | 12537 (74.9) | 937 (32.6) | 8433 (68.6) |
| **AKI** |  |  |  |  |  |
| **Stage1** | 1479 (11.8) | 2469 (22.9) | 1624 (9.7) | 1113 (38.8) | 2336 (19.0) |
| **Stage2** | 862 (6.9) | 1289 (12.0) | 2462 (14.7) | 544 (18.9) | 1044 (8.5) |
| **Stage3** | 773 (6.2) | 407 (3.8) | 109 (0.7) | 278 (9.7) | 486 (4.0) |

MMH, MacKay Memorial Hospital; NCKU, National Cheng Kung University Hospital; TCVGH, Taichung Veterans General Hospital; TVGH, Taipei Veterans General Hospital; KMUH, Kaohsiung Medical University Hospital; AKI, acute kidney injury.

Data are presented as number (%).

# Supplement figure 1 Patient selection for model development in the TCVGH derivation cohort


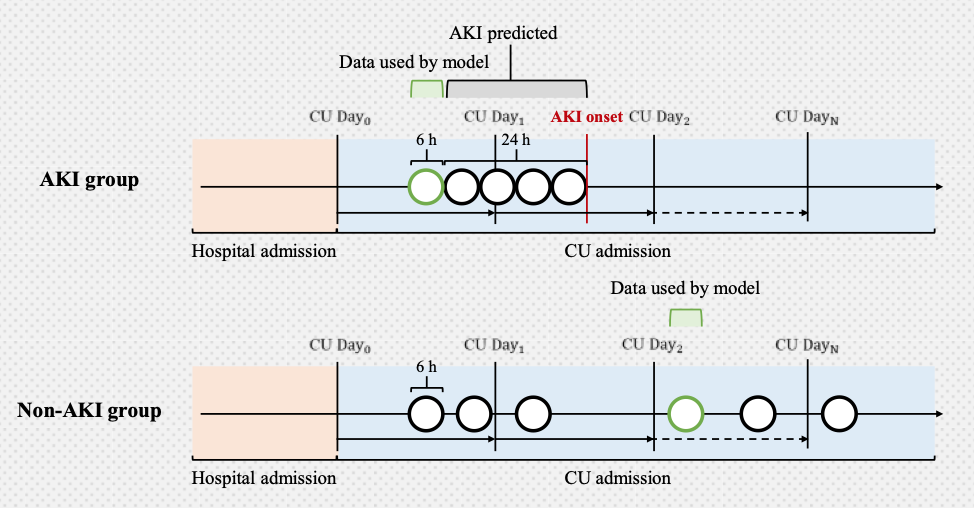


# Supplement figure 2 Study design of the feature window and prediction window for machine learning AKI prediction


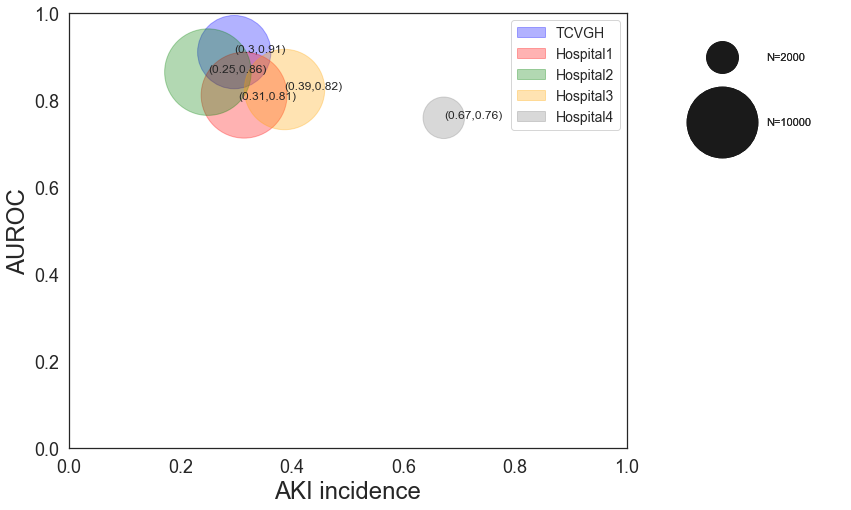


# Supplement figure 3 The association of AKI prevalence, case number, and model performance in a visualized graph
